# Reactive Extraction of Malic Acid using Trioctylamine in 1–Decanol: Equilibrium Studies by Response Surface Methodology Using Box Behnken Optimization Technique

**DOI:** 10.1038/s41598-020-59273-z

**Published:** 2020-02-12

**Authors:** Victoria Inyang, David Lokhat

**Affiliations:** Discipline of Chemical Engineering, University of KwaZulu-Natal, Howard College Campus, Durban, South Africa

**Keywords:** Engineering, Chemical engineering

## Abstract

Reactive extraction is a significant technique employed for the removal of organic acids such as carboxylic acid which are usually present in low concentrations in aqueous solutions. This technique was explored by applying Response Surface Methodology (RSM) in process parameter optimization for malic acid recovery from aqueous streams using Trioctylamine as extractant and 1-decanol as organic diluent. Malic acid, a C_4_ dicarboxylic acid has a wide variety of applications in the polymer, food, chemical and pharmaceutical industries. The optimization of the response function: extraction efficiency was systematically carried out using three process parameters for reactive extraction: temperature, initial malic acid concentration and extractant (Trioctylamine) composition. Response Surface Methodology in combination with Box-Behnken design involving seventeen experimental runs was employed for malic acid reactive extraction in this study. A statistical second-order polynomial predicted an extraction efficiency of 97.53%. The optimum conditions of the process variables were found to be: temperature: 304.73 K, acid concentration: 0.25 kmol/m^3^, Trioctylamine composition: 23.54% (v/v). Under these optimum conditions, the experimental response of extraction efficiency of 93.25% was obtained. The experimental results obtained was in close conformity with the predicted values by numerical optimization using Response Surface Methodology. These findings can pave the way for the reactive separation process design for recovery of carboxylic acids from dilute aqueous waste streams as well as a fermentation broth.

## Introduction

Carboxylic acids are usually present in low concentrations in aqueous stream. Interest in the recovery of these acids from dilute aqueous solutions with acid concentrations lower than 10% (w/w), has received considerable attention^1^. Most recent research is targeted at carboxylic acid separation process selection with low material consumption and less energy requirement in the downstream processing. An intensified process that satisfies these requirements is reactive separation since significant improvements are achieved in both stages of reaction and separation. Carboxylic acids such as butyric acid, lactic acid, propionic acid, malic acid are useful bulk chemicals for several industries. Malic acid is a C_4_-dicarboxylic acid and also an intermediate of the tricarboxylic acid cycle. It has a variety of applications in polymer, food, chemical and pharmaceutical industries^2^. The downstream recovery technique for carboxylic acids accounts for 30–50% of the overall production cost^3,4^. Hence, the current interest in finding a more cost-effective recovery technique. Reactive extraction is a significant technique for the separation of important carboxylic acids which leads to a high solute distribution coefficient as a result of combining physical and chemical phenomena^5^. The factors that are favourable for carboxylic acids reactive extraction include an existing functional group which increases capacity and selectivity in solute molecules, a high driving force of complexing agents as a result of low concentration and low volatility of the solute^6^. The application of reactive extraction to different carboxylic acid from dilute aqueous solution has been successfully carried out; such as; Latic acid^7,8^, Itaconic acid^9,10^ Succinic acid^11,12^, Levulinic acid^13,14^, pyruvic acid^15^ tartaric acid^16^, propionic acid^17–19^. The appropriate solvent selection as constituents of the organic phase is an underlining factor for high distribution coefficient and extraction efficiency. High viscous extractants for example phosphorus bonded, oxygen-bearing and hydrocarbon, high molecular weight aliphatic amines with diluents are often employed in the carboxylic acid reactive extraction process for improvement of physical properties such as interfacial surface tension and viscosity^1,20^.

In general; there are primary, secondary and tertiary amines in amine-type extractants. Among these amines, the tertiary amines offer better advantage in reactive extraction because the primary and secondary amines tend to react irreversibly with carboxylic acids and therefore, the stripping of solvent becomes difficult^21^. Reactive extraction using long-chain aliphatic tertiary amines (anion exchange extractants such as trioctylamine) with seven to nine carbon atoms in each alkyl group has been studied as the most effective, efficient and widely employed extractants for carboxylic acids. When dissolved in different modifiers (solvents), they are powerful extractant reagents for the carboxylic acids^22^. They provide high extraction efficiency (>90%) and are less expensive as compared with the oxygen donor or phosphorus-based extractants^8^. These extractants used in the reactive extraction processes in organic acid separation can be recycled thus making them effective and efficient. Ratchford, *et al*.^23^ studied the effects of amine structure and the solvent properties. The solvation of the whole amine-acid complex is based on dipole-dipole interaction and has been found to play a key role in the neutralization reaction between acid and amines. Amine-based extractants are highly favourable for carboxylic acid extraction, for example, the citric acid process was technically feasible using tertiary amine extractant^24^. In addition to high efficiency and selectivity, they provide for product concentration through extraction at about ambient temperatures. These extractants are often used with an organic solvent as diluent which has a significant effect on the extraction performance, acid loading and stoichiometric association^25–28^. The diluent may consist of one or more components, inert or active. Various active polar and proton or electron-donating diluents (halogenated aliphatic/aromatic hydrocarbons, ketones, nitrobenzenes, higher alcohols), enhance the extraction. On the other hand, inert diluents (long-chain paraffin, benzene etc.), limit the extractant capacity^10,29,30^. Significantly, diluents with a moderate polarity such as a long chain or higher alcohols (for example 1-octanol and 1-decanol) greatly improves the solvation power of the acid-amine complex. They also influence the basicity of the amine and improve phase separation and the stability of the ion-pair (acid-amine complex) formed. Thus preventing third phase formation which limits the extraction ability giving a high distribution coefficient^8^. Significant research studies have been conducted on the influence of diluents on amine extractants in carboxylic acids recovery^26,28,31,32^. With this background, 1-decanol with moderate polarity, water-insoluble diluent and less toxic has been considered in the present study. And so, TOA in combination with 1-decanol has been chosen for this work as effective extractant and diluent respectively.

Several studies have been conducted on the reactive extraction process on organic acid separation from fermentation aqueous waste stream. However, studies which employ statistical technique on the experimental design of malic acid recovery from aqueous solution using an intensified process such as reactive liquid-liquid separation process is limited in the literature. The selection of a suitable technique for evaluating different process parameter is important as well as any interactions involved while minimizing the number of experimental runs. This research study is centered on the application of Box Behnken in data analysis, optimizing process parameters and exploring appropriate conditions to be employed in the reactive extraction process for optimal extraction efficiency and distribution coefficient.

**Figure 1:**
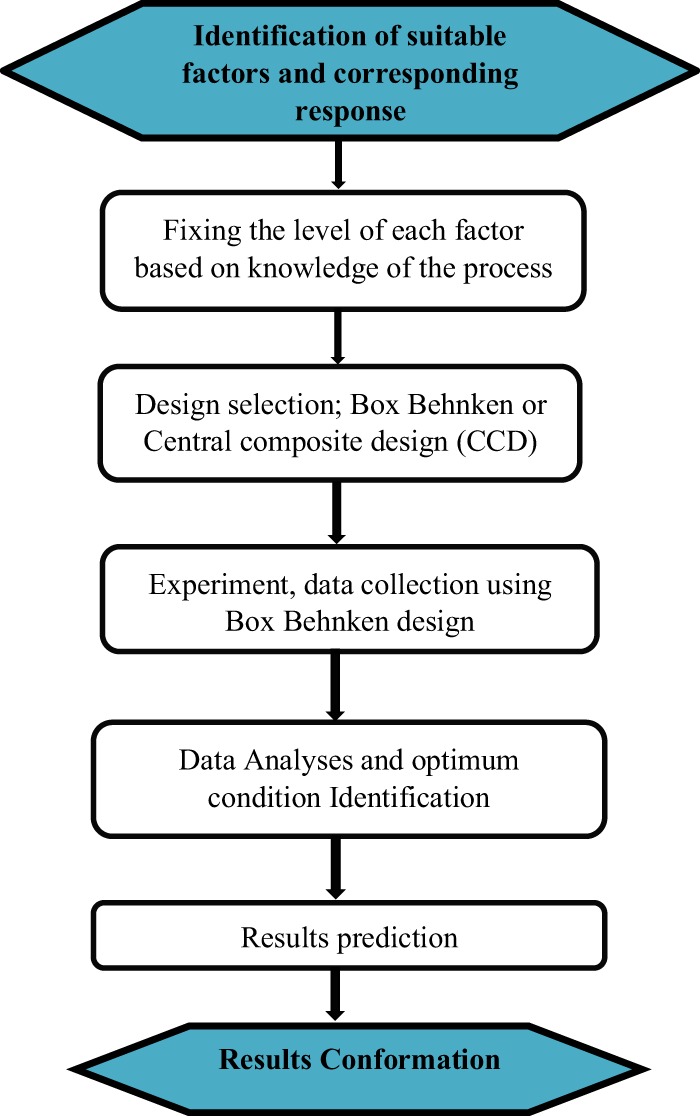
RSM step-wise procedures adopted in the study.

The study intends to enhance the extraction yield for effective and efficient recovery of malic acid from dilute aqueous solution by employing trioctylamine as extractant mixed with high polarity solvent,1-decanol as diluent. Also, the major factor influencing the reactive extraction process include temperature, the concentration of the extractant and acid. This work is also aimed at analysing, optimizing and finding appropriate conditions of these process variables using response surface method (RSM) by employing a Box-Behnken design to maximize the efficiency of the malic acid reactive extraction process (% E). The statistical design of experiment is used for process parameter optimization for the malic acid extraction to prevent drawbacks obtained from classical methods. The optimum parameters of the intensified reactive extraction process will be used to determine the reaction rate kinetics of the extraction process. Response surface methodology (RSM) is an effective tool for statistical design of experiments, model development and for finding complex processes to optimize the target yield (s). It is also a statistical tool used to create a link between a set of defined experimental variables and the observed results. The adopted stepwise procedure in this study is as shown (Fig. 1).

## Results and Discussion

The experimental data was analyzed using Response Surface Methodology (RSM) to examine the combined effects of temperature, malic acid concentration and TOA composition and the results are reported (Table 1). Analysis of Variance (ANOVA) was employed to predict the correlation between independent process variables and the corresponding responses and a second-order polynomial equation was obtained for extraction efficiency of malic acid and is presented in “Eq. (1)”.

1 $${\rm{E}} \% =79.28+6.63{X}_{1}+7.93{X}_{2}-24.4{X}_{3}+1.18{X}_{1}{X}_{2}+4.15{X}_{1}{X}_{3}+7.35{X}_{2}{X}_{2}-12.12{X}_{1}^{2}-11.16{X}_{2}^{2}+5.94{X}_{3}^{2}$$

where the variable ${X}_{1}$ = temperature, ${X}_{2}$ = Acid concentration and ${X}_{3}$ = TOA concentration.

**Table 1:** Experimental design of variables (coded) for malic acid extraction efficiency E%.

Std	Run	Factor 1	Factor 2	Factor 3	Response 1
		Temperature	TOA Composition	Acid Concentration	Extraction Efficiency
		K	%v/v	kmol/m^3^	%E
10	1	0.000	1.000	−1.000	98.571
1	2	−1.000	−1.000	0.000	39.307
3	3	−1.000	1.000	0.000	53.030
16	4	0.000	0.000	0.000	79.264
7	5	−1.000	0.000	1.000	40.750
9	6	0.000	−1.000	−1.000	97.619
5	7	−1.000	0.000	−1.000	98.571
11	8	0.000	−1.000	1.000	34.833
14	9	0.000	0.000	0.000	79.307
17	10	0.000	0.000	0.000	79.394
4	11	1.000	1.000	0.000	75.030
12	12	0.000	1.000	1.000	65.191
6	13	1.000	0.000	−1.000	97.143
13	14	0.000	0.000	0.000	79.091
2	15	1.000	−1.000	0.000	56.606
8	16	1.000	0.000	1.000	55.917
15	17	0.000	0.000	0.000	79.351

**Figure 2:**
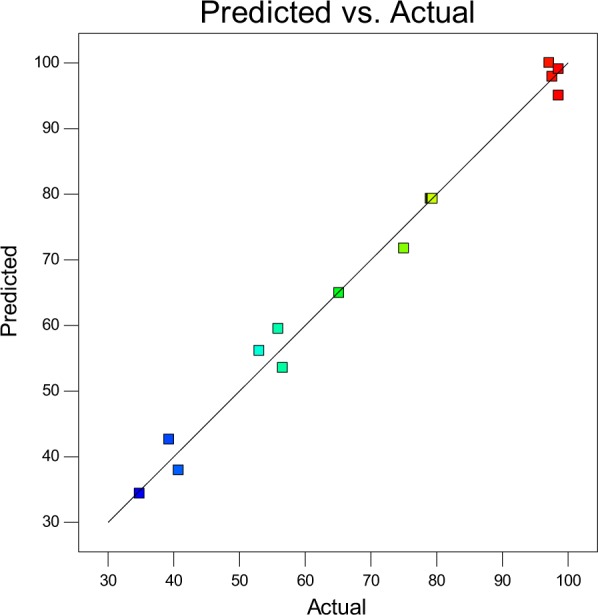
Predicted model plot against experimental extraction efficiency (%E) from RSM Model.

The regression model and ANOVA of extraction efficiency for reactive extraction of malic acid using TOA in 1-decanol are presented (Table 2). From the Table, the model F-value of 67.66 indicates the model is significant. There is only a 0.01% chance that an F-value this large could occur due to noise. Values of “Prob > F” less than 0.0500 indicate model terms are significant. In this case, ${X}_{1}$, ${X}_{2}$, ${X}_{3}$, $\,{X}_{1}^{2}$, ${X}_{2}^{2}$, ${X}_{3}^{2}$ are significant model terms. Also, values of “Prob > F” greater than 0.1000 indicate the model terms are measured as insignificant. The coefficient of correlation with lower P values are considered to to be more important. The correlation coefficient values (R2) was adequate (0.9886) for the response (P ≤ 0.05)^33^. The plots of the correlation coefficient and the adjusted values for comparing the model fitness is presented (Fig. 2). The adequate precision value which measures single to noise ratio is expected to be greater than 4. The model ratio gotten in this work is 24.866 which indicates an acceptable signal and can be employed to pilot the design space. The variation coefficient obtained is relatively low (CV = 4.84%), which indicates the accuracy and reliability of the model. The contour plots and three dimensional (3D) graphs were generated as a result of the effects of the interaction between two independent variables on the response by maintaining one constant process variables at zero levels (coded**)**^34^.

**Table 2:** Analysis of variance and response surface regression model for malic acid reactive extraction.

Source	Sum of	df	Mean	F	p-value	
	Squares		Square	Value	Prob > F	
Model	7207.38	9	800.82	67.66	<0.0001	significant
*X*_1_-Temperature	351.61	1	351.61	29.71	0.0010	
*X*_2_-TOA Concentration	503.34	1	503.34	42.53	0.0003	
*X*_3_-Acid Concentration	4763.57	1	4763.57	402.49	<0.0001	
*X*_1_*X*_2_	5.53	1	5.53	0.47	0.5164	
*X*_1_*X*_3_	68.85	1	68.85	5.82	0.0466	
*X*_2_*X*_3_	216.16	1	216.16	18.26	0.0037	
*X*_1_^2^	618.82	1	618.82	52.29	0.0002	
*X*_2_^2^	524.85	1	524.85	44.35	0.0003	
*X*_3_^2^	148.41	1	148.41	12.54	0.0095	
Residual	82.85	7	11.84			
Lack of Fit	82.79	3	27.60	2017.72	<0.0001	significant
Pure Error	0.055	4	0.014			
Cor Total	7290.23	16				
Std.Dev = 3.44 R-Squared = 0.9886						
Mean = 71.12 Adj R-Squared = 0.9740						
C.V.% = 4.84 Adeq Precision = 24.866

### Effect of the different variables on reactive extraction of malic acid

In order to access the effect of the different process variables on the extraction efficiency, experimental design of the single factor effect was adopted (one variable at a time). The three variables considered were temperature, initial acid concentration and trioctylamine composition. The experimental design was achieved by vaying one variable while holding the other two independent variable constant (see supplementary Table S1 and Figs. S1–S3).

**Figure 3:**
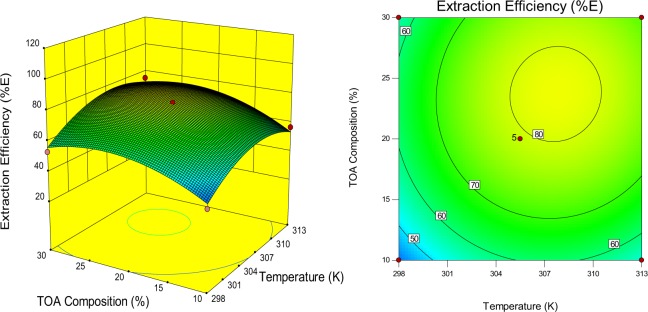
Response surface plot and a contour-lines map displaying the effects of interaction between TOA composition and temperature variables on extraction efficiency.

**Figure 4:**
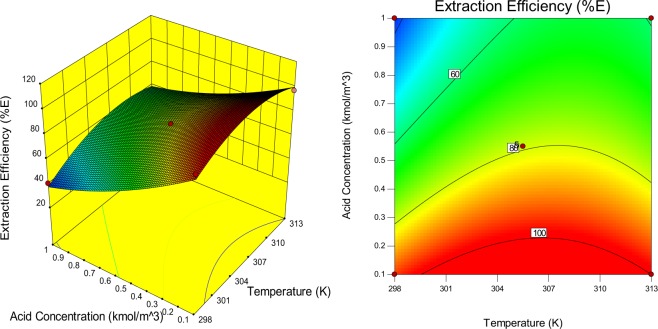
Response surface plot and a contour-lines map displaying the effects of interaction between acid concentration and temperature variables on extraction efficiency.

**Figure 5:**
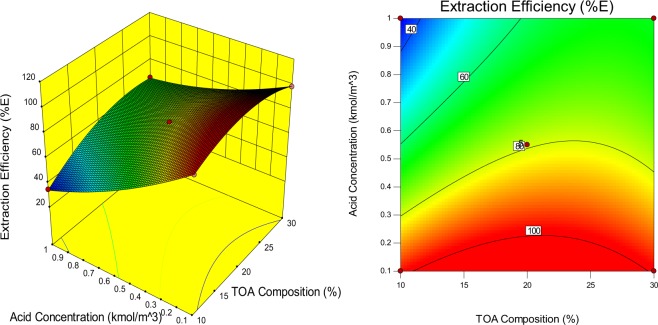
Response surface plot and a contour-lines map displaying the effects of interaction between acid concentration and TOA composition variables on extraction efficiency.

The models selected has explained the combined effect of three variables on the malic acid extraction efficiency (%E). The three dimensional (3D) graphs of the response surfaces and the corresponding contour lines map were necessary to explain the effects of the different process parameters as presented (Figs. 3, 4, 5). The experimental results illustrated (Fig. 3), presents the effects on the interaction between TOA composition and temperature on the extraction efficiency. It can be interpreted from the figure that extraction efficiency increases with an increase in extractant concentration (10–30%, v/v) irrespective of the temperature. This might be a product of the increase in extractant concentration which increases the extractability of the extractant and further helps to reach extraction equilibrium and the ability to form a complex^35^. The complexation reactions at equilibrium occur at the organic-aqueous interface and is an exothermic process. The malic acid-TOA complex brings about orderliness in the reactive extraction process thus decreasing the randomness and entropy of the system. The temperature increase also increases the kinetic energy of the molecules thereby interrupting certain interactions and acid-amine molecule combinations to form a stable complex at the interface^36^. Another reason could be as a result of the exothermic reactions of the transfer of proton and formation of hydrogen bond which decreases the system entropy^30^.

The interactive effects of malic acid concentration and temperature on the extraction efficiency is elucidated (Fig. 4). It can be interpreted from the figure that the extraction efficiency decreased with an increase in malic acid concentration regardless of the temperature. This is because the tendency of extractant overloading increases with solute concentration^37^. The extraction efficiency decreases with a rise in acid concentration and on further temperature enhancement. This might be due to the disturbance of extractant and solute molecules interaction in the organic phase with thermal energy increase leading to a reduction in the formation of the complex^38^.

The extraction efficiency at a higher solvent ratio (30%, v/v) initially increases up to a certain point and then starts decreasing with further increase in acid concentration (Fig. 5). This might be because of the presence of 1-decanol, a polar organic solvent being reduced with an increase in extractant ratio owing to H-bonding between C=O of the acid-extractant complex and the proton of the polar diluent^39^.

### Process variables optimization

The same software, design expert was employed in the numerical optimization of the process parameters to maximize the malic acid extraction efficiency. The predicted maximum values obtained from the model for the reactive extraction of malic acid for maximum extraction efficiency are temperature: 304.73 K, acid concentration: 0.247 kmol/m^3^, Trioctylamine composition: 23.54% (v/v) and the model prediction for the extraction efficiency under these optimum conditions as 97.53%. Experimental verification and validation were conducted at the optimized process variables and the results obtained (93.25%) were in good agreement with the predicted model response values.

## Conclusion

In this study, the reactive extraction of malic acid onto a biphasic organic-aqueous system with trioctylamine in 1-decanol as extractant and diluent respectively for determining the extraction efficiency and distribution coefficient was successfully carried out. Box Behnken design was employed to study the interactive effects of different process parameters on the equilibrium studies of malic acid. The regression analysis using ANOVA, experimental design and quadratic model was developed to predict and optimize the functional relationship between the process variables (temperature, malic acid concentration and extractant composition) and the response (extraction efficiency and distribution coefficient). The optimum conditions of the process variables were found to be: temperature: 304.73 K, acid concentration: 0.247 kmol/m^3^, Trioctylamine composition: 23.54% (v/v). Under these optimum conditions, the experimental value for the extraction efficiency (E%) was 93.25%. These findings and results can pave the way for the reactive separation process design for recovery of carboxylic acids from dilute aqueous waste streams as well as a fermentation broth. This can unlock pathways to the recovery of these acids in low concentrations.

## Materials and Methods

### Experimental chemicals deployed

All the chemicals, Malic acid (C_4_H_6_O_5_), density 1.61 g/mL, Trioctylamine (TOA), [CH_3_(CH_2_)_7_]_3_N, density 0.809 g/mL and 1-decanol, density 0.829 g/mL deployed in this study at purity of 98% were purchased from Sigma-Aldrich and the experimental water was obtained using an Elga PURELAB Option Q purification system. Deionized water from our laboratory was used throughout the experiment. Phenolphthalein indicator and 0.1 M Sodium hydroxide were also purchased from Sigma-Aldrich. All chemicals were used as supplied with no further purification.

### Equilibrium studies

Reactive extraction equilibrium studies were conducted by preparing 25 mL aqueous solution (0.1 kmol/m^3^ concentration of malic acid) and 25 mL organic phase solution was prepared by mixing (10–30)% of Trioctylamine extractant (%v/v) equivalent to (0.229–0.687 kmol/m^3^) in 1- decanol at temperatures between (298–313 K) in an orbital shaker which was placed in the oven for 5 hours at 120 rpm, the two phases was kept to settle for 2 hours. The aqueous phase analysis was carried out using the titration method with 0.1N NaOH and phenolphthalein as an indicator to obtain malic acid concentration. The organic phase concentration of malic acid was determined through mass balance. The experimental runs were done in triplicate to confirm the reproducibility of results.

### Experimental design and response surface methodology

The different experimental cycles were carried out using the design of experiment template obtained using Box-Behnken design (BBD) with three variables at three levels each in the optimization of the process variables used in the reactive extraction process. The Design-Expert version 10.0 (Statease Inc., Minneapolis, USA) was employed in this study. The un-coded (original) value of the different factors and their corresponding coded levels employed in the experimental design is as shown (Table 3).

**Table 3:** Range of different variables for reactive extraction of Malic acid.

Factors	Units	Coded Values	Level	High
			Low	
Temperature	K	Factor X_1_	298	313
Solvent Composition (v/v)	%	Factor X_2_	10	30
Acid Concentration	kmol/m^3^	Factor X_3_	0.100	1.000

There are three main stages in Response Surface Methodology which include:

i. Parameter selection and experimental design; finding a suitable estimation between independent process (factors) and the dependent (response) variables^34^.
ii. Modeling of the response obtained from experimental results through regression and analysis of variance.
iii. Response optimization; the optimum values of the independent process parameters; malic acid concentration, extractant ratio (%v/v) and temperature (K) were estimated to gain maximum value of the response, Extraction Efficiency (E%).

The relationship established between coded (x_i_) and real (X_i_) value is represented in “Eq. (2)” as

2 $${\rm{Coded}}\,{\rm{value}}\,({{\rm{x}}}_{{\rm{i}}})=\frac{{X}_{i}-{X}_{0}}{\Delta {X}_{i}},{\rm{i}}=1,2,\mathrm{3.....}.,{\rm{n}}$$

where ΔX is the phase change and X_0_ is the real value at the center position

Taking into consideration all interactions of the input parameters (linear to linear and linear to quadratic), the behavior of the system can be described by “Eq. (3)”.

3 $${\rm{Y}}={\beta }_{0}+{\sum }_{i=1}^{n}{\beta }_{i}{X}_{i}{\sum }_{i=1}^{n}{\beta }_{ii}{X}_{i}^{2}+{\sum }_{i < j}^{n}{\sum }_{j}^{n}{\beta }_{ij}{X}_{i}{X}_{j}\,+{\rm{\varepsilon }}$$

where I and j are linear and quadratic coefficients respectively; n is the number of experimental parameters (n = 3). ${\beta }_{0}$ is constant coefficient, ${\beta }_{i}$ is the linear outcome or slope of input factor, ${\beta }_{ii}{X}_{i}$ is a quadratic outcome of input factor ${X}_{i}$ and ${\beta }_{ij}$ is linear effect interaction between input factors ${X}_{i}$ and ${X}_{j}$, ε is the residual error.

### Theory of reactive extraction of Malic acid

The reactive extraction process for malic acid accounts for the combined effect of physical and chemical extraction. Physical extraction (dimerization and ionization) involves solute separation which is free of complexities. In physical extraction, factors responsible and considered according to some researchers^9,18,40^ are:

- Aqueous phase ionization of Malic acid (as H^+^A^−^) and its corresponding non-facilitated transportation to the organic phase.
  4 $$\begin{array}{rcl}{[HA]}_{aq} & \leftrightarrow  & {{\rm{A}}}^{-}\,+\,{{\rm{H}}}^{+}\\ Un \mbox{-} dissociated\,Malic\,acid &  & Dissociated\,Malic\,acid\end{array}$$
- Equilibrium distribution and partial malic acid dissociation between phases
  5 $$\begin{array}{rcl}{[HA]}_{aq} & \leftrightarrow  & {[HA]}_{org}\\ Un \mbox{-} dissociated\,Malic\,acid &  & Dissociated\,Malic\,acid\end{array}$$
- Organic phase acid dimerization

6 $$2{[HA]}_{aq}\leftrightarrow {[HA]}_{org}^{2}$$

Distribution coefficient is presented with respect to dimerization coefficients according to^41^:

7 $$\begin{array}{rcl}{K}_{D}^{diluent} & = & P+2{P}^{2}D{[HA]}_{aq}\\ {\rm{where}}\,P & = & {[HA]}_{org}/{[HA]}_{aq}\,D={[HA]}_{2,org}/{[HA\}}_{org}^{2}\end{array}$$

In chemical or reactive extraction (diffusion and solubility) of Malic acid, the process involved the contacting of a second phase extractant (trioctylamine and 1-decanol) that will reversibly react with the solute. The reaction with the liquid-liquid extraction which is attained in one unit operation may be interpreted in three steps. Initially, transportation of reactants to the interface forming aqueous-organic phase interface from the bulk and interaction with the molecules of extractant, thus the formation of extractant-acid complex and finally, the complex that is formed is transported unto the organic phase for removal of the malic acid^42^. To explain and describe the mechanism of chemical extraction using “Eq. (9)”, the equilibrium constant according to^9^ is presented in Equation as:

8 $$Solute+n.Extractant\leftrightarrow Complex$$

9 $${K}_{c}=\frac{[Complex]}{[Solute]{[Extractant]}^{n}}$$

During the reactive extraction of malic acid, the three mechanisms of reaction involved (anion exchange, the formation of ion-pair and H-bond) were noted due to the acid-extractant complex formation in the solution dependent upon trioctylamine (extractant) basicity and the constant of dissociation of the extracted species^20^. The complex formed will remain at the interface otherwise it will orient towards the interphase with any hydrophilic complexes formed in the organic phase^43^. When there exists any non-dissociated form of malic acid in the aqueous phase, the malic acid reaction with trioctylamine takes place through the formation of hydrogen bond leading to trioctylamine-malic acid complex formation of (1:1) and also (1:2), (1:3) at higher concentration of malic acid.

10 $$\begin{array}{c}{R}_{3}N+HA\leftrightarrow {R}_{3}N-HA\\ TOA\,MA\,TOA-MA\,complex\end{array}$$

This extracted acid by the amine extractant is generally known as an ammonium salt. The ion-pair formation complex occurs when Malic acid exists in the dilute aqueous phase in its dissociation form. The extent of association of the ion pair (acid radical and alkylammonium radical) leads to the quantification of extraction degree and stability^6^.

11 $$\begin{array}{lllll}{R}_{3}N & + & {H}^{+}+{A}^{-} & \leftrightarrow  & {R}_{3}N{H}^{+}{A}^{-}\\ TOA &  & Malic\,acid &  & TOA-MAcomplex\\  &  & (dissociated\,form) &  & \end{array}$$

The hydrogen bond formation could also be possible between the complex C = O of (1:1) trioctylamine-malic acid with 1-decanol which is present as a diluent in the oil phase^39^.

12 $$\begin{array}{lllll}{R}_{3}N{H}^{+}{A}^{-} & + & {{\rm{C}}}_{10}{H}_{21}OH & \leftrightarrow  & {R}_{3}N{H}^{+}{A}^{-}\,{H}^{+}{{\rm{OC}}}_{10}{{\rm{H}}}_{21}\\ TOA\,-\,Acid\,complex &  & 1 \mbox{-} Decanol &  & TOA \mbox{-} Acid \mbox{-} {1}\,Decanol\,Complex\end{array}$$

The malic acid reactive extraction process performance was assessed by the Extraction efficiency and Distribution coefficient (K_D_) at equilibrium as the ratio of the concentration of acid in the organic phase ${[HA]}_{org}$ to the concentration in aqueous phase ${[HA]}_{aq}$ as shown in “Eq. (13)” and “Eq. (14)”.

13 $${\rm{Distribution}}\,{\rm{coefficient}}\,({{\rm{K}}}_{{\rm{D}}})=\frac{{[HA]}_{org}}{{[HA]}_{aq}\,}$$

14 $${\rm{Extraction}}\,{\rm{efficiency}}\,( \% {\rm{E}})=\frac{{K}_{D}\,\times \,100}{1+{K}_{D}}$$

## Supplementary information

Supplementary information.
