# Supplementary material for: Reactive Extraction of Malic Acid using Trioctylamine in 1–Decanol: Equilibrium Studies by Response Surface Methodology Using Box Behnken Optimization Technique
Source: Sci Rep. 2020 Feb 12;10:2400. doi: 10.1038/s41598-020-59273-z (PMC7016146; doi:10.1038/s41598-020-59273-z)
Supplement: Supplementary file 1 — Supplementary information. [file 41598_2020_59273_MOESM1_ESM.pdf]

Reactive Extraction of Malic Acid using Trioctylamine in 1–Decanol: Equilibrium Studies by Response Surface Methodology Using Box Behnken Optimization Technique

Inyang, Victoria[1*] and Lokhat, David[2+]

[1,2]Discipline of Chemical Engineering, University of KwaZulu-Natal, Howard College Campus, Durban, South Africa.

*Correspondence and requests for materials should be addressed to I.V. (email: vickyinyang@yahoo.com)

	Factor 1	Factor 2	Factor 3	Response
Run :	Temperature	B: TOA Concentration	C: Acid Concentration	Extraction Efficiency
	K	%	kmol/m3	%E
1	298	20	1	40.7500
2	305.5	20	1	48.1667
3	313	20	1	55.9167
4	313	10	0.55	56.6061
5	313	20	0.55	66.1905
6	313	30	0.55	75.0303
7	305.5	0.1	30	98.5714
8	305.5	0.55	30	89.6537
9	305.5	1	30	65.1905

Table S1. Experimental design for single factor effects.

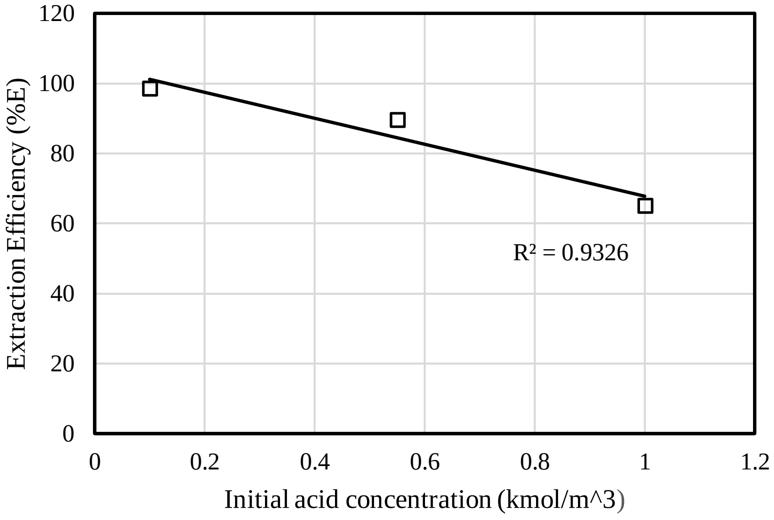

Figure S1. Effect of initial acid concentration on extraction efficiency at 30 (%v/v) trioctylamine composition and305.5K.

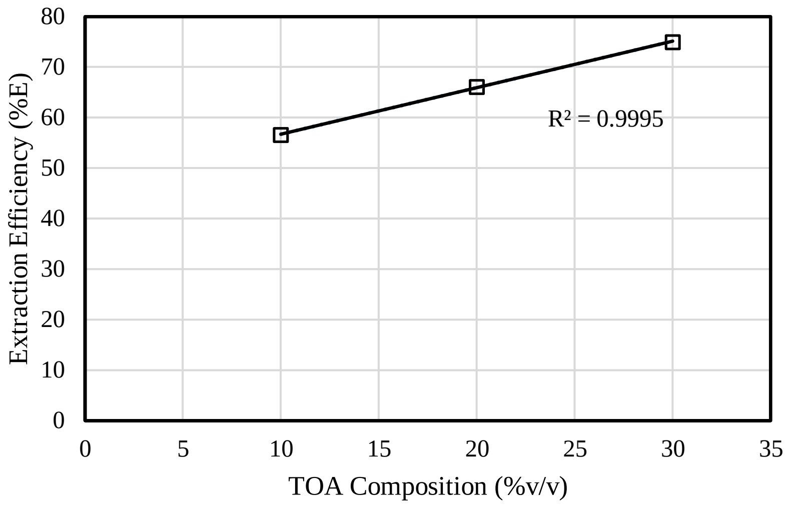

Figure S2. Effect of trioctylamine composition (%v/v) on the extraction efficiency at initial acid concentration of 0.55 kmol/m^3and temperature of 313K.

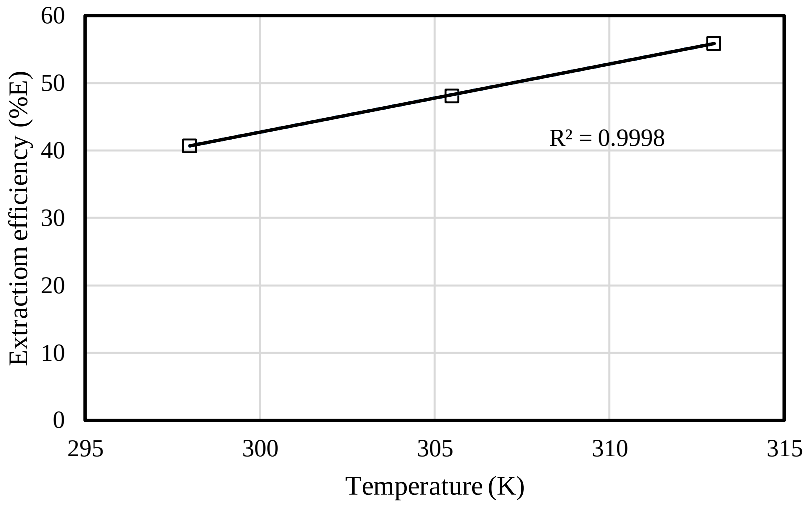

Figure S3. Effect of temperature on extraction efficiency (%E) at initial concentration of 1 kmol/m^3 and 20 (%v/v) trioctylamine.
